# Morphological and community changes of turf algae in competition with corals

**DOI:** 10.1038/srep12814

**Published:** 2015-08-05

**Authors:** Neidy P. Cetz-Navarro, Lizette I. Quan-Young, Julio Espinoza-Avalos

**Affiliations:** 1 ECOSUR, Avenida Centenario km 5.5, Colonia Pacto Obrero Campesino, Chetumal 77014, Quintana Roo, Mexico; 2 Posgrado en Oceanografía Costera, Instituto de Investigaciones Oceanológicas-Facultad de Ciencias Marinas, Universidad Autónoma de Baja California, Apdo. Postal 453, km 103 Carretera Tijuana-Ensenada, Ensenada 22860, Baja California, Mexico

## Abstract

The morphological plasticity and community responses of algae competing with corals have not been assessed. We evaluated eight morphological characters of four species of stoloniferous clonal filamentous turf algae (FTA), including *Lophosiphonia cristata* (*Lc*) and *Polysiphonia scopulorum* var. *villum* (*Psv*), and the composition and number of turf algae (TA) in competition for space with the coral *Orbicella* spp. under experimental and non-manipulated conditions. All FTA exhibited morphological responses, such as increasing the formation of new ramets (except for *Psv* when competing with *O. faveolata*). Opposite responses in the space between erect axes were found when *Psv* competed with *O. faveolata* and when *Lc* competed with *O. annularis*. The characters modified by each FTA species, and the number and composition of TA species growing next to coral tissue differed from that of the TA growing at ≥3 cm. The specific and community responses indicate that some species of TA can actively colonise coral tissue and that fundamental competitive interactions between the two types of organisms occur within the first millimetres of the coral−algal boundary. These findings suggest that the morphological plasticity, high number, and functional redundancy of stoloniferous TA species favour their colonisation of coral tissue and resistance against coral invasion.

Coral reefs are being degraded around the world^1^, and those of the Caribbean are among the most affected^2^. Turf algae (TA) and macroalgae are significant assemblages on degraded reefs, and the greater interaction between corals and algae under these conditions is part of a negative feedback mechanism that restricts reef recovery^3^; however, the direct involvement of algae in coral damage and coral mortality and the potential mechanisms used by algae to invade the coral tissue remain uncertain^4^. In general, it is assumed that corals must be injured, bleached, stressed, or dead before algae can colonise them^5^,^6^,^7^.

The relatively passive role that algae are assumed to play in colonising corals contrasts with their high morphological plasticity in biological interactions. Algal morphology is modified as a response to physical, chemical, and biological factors^8^,^9^,^10^,^11^,^12^. It has also been suggested that algal phenotypic plasticity may be a key mechanism involved in the occupation of different ecological niches^13^ and in the invasion of species^14^, as observed in terrestrial clonal plants. For example, vascular clonal plants show morphological changes in their search for resources in spatially heterogeneous environments^15^,^16^ during fertilisation^17^, in response to disturbances (conferring a survival advantage)^18^, and in competitive interactions^19^. Fordyce^20^ concluded that ecological interactions mediated by phenotypic plasticity are common in nature, and the results and the intensity of those interactions are mediated by the morphological responses of the interacting organisms.

TA constitute the most abundant algal assemblage on Caribbean reefs, frequently growing in contact with the tissue of *Orbicella* spp. (*Orbicella* = *Montastraea annularis* species complex)^21^, one of the most important reef-building coral genera in the Caribbean^22^. When these two organisms are in contact, TA stress, compete with, and overgrow *Orbicella* spp.^23^,^24^. Typical or stoloniferous clonal algal species (attached to the substratum at several points through rhizoids from a prostrate axis, along which short and branched erect axes rise^25^,^26^) are commonly found within the TA assemblage.

The aim of this study was to determine the morphological plasticity and changes in the percentage of genets undergoing reproduction of four species of stoloniferous clonal filamentous turf algae (FTA): two under experimental conditions (reciprocal transplantation of healthy and dead coral cores) and three (including one of the first two) under non-manipulated conditions (growing in three dead coral zones close to live coral tissue). In this study, a genet is defined as a fragment consisting of a prostrate axis with several erect axes and a ramet as an erect axis that adopts the morphology of a prostrate axis with additional erect axes and rhizoids. The community of TA competing with *Orbicella* spp. was also evaluated for species composition, species number, and percentage of species reproducing. Our results show that TA competing with the coral tissue of *Orbicella* spp. respond at both the species and community levels, and these changes suggest that adaptive algal plasticity in morphology and community responses may impede coral defences against algal colonisation, that not all TA species are adapted to compete successfully for space with *Orbicella* spp., and that important coral−algal interactions occur within 0.5 cm of the coral−algal boundary.

## Results

### 1) Turf algal species richness under non-manipulated and manipulated conditions

#### a) Non-manipulated conditions

The assemblages of TA growing in three zones in relation to *O. annularis* (the primary front, PF: the first 0.5 cm next to *O. annularis* tissue; the secondary front, SF: a 1-cm-wide band located 3–4 cm from coral tissue; and the rearguard zone, RE: ≥30 cm from the periphery of coral tissue) were composed of 96 taxa, with rhodophytes being the most abundant (55 taxa (57%)) than the other groups of autotrophs (18 Chlorophyta (19%), 10 Phaeophyceae (10%) and 13 Cyanobacteria (14%)) (see Supplementary Table S1).

#### b) Experimental conditions

The assemblages of TA growing on transplanted *Orbicella faveolata* cores were composed of 86 taxa, with rhodophytes being more abundant (51 taxa (59%)) than the other groups of autotrophs (15 Chlorophyta (18%), 14 Phaeophyceae (16%) and 6 Cyanobacteria (7%)) (see Supplementary Table S4).

### 2) Response of turf algal communities under non-manipulated and manipulated conditions.

#### a) Non-manipulated conditions:

Composition of turf algal communities, number of species, and percentage of species undergoing reproduction. The general composition of TA species in the PF differed from that in the SF and RE (pseudo-F_2,27_ = 1.98, p < 0.05). Five taxa (3 Rhodophyta, 1 Chlorophyta, and 1 Cyanobacteria) contributed the most to the differences between zones (SIMPER): *Anotrichium tenue* was more frequent, whereas *Taenioma nanum*, *Bryobesia johannae,* and *Lyngbya sordida* were less frequent in the PF than in the SF and RE. *Champia parvula* var. *prostrata* was absent in samples from the PF but was a representative species of the SF and RE. Some species were found exclusively in one of the three zones: 6 in PF, 8 in SF, and 19 in RE (see Supplementary Table S1).

The average number of TA species was lower in the PF (20) than in the RE (30) (F_2,27_ = 11.35, p < 0.05). The percentages of species with reproductive structures were similar in all three zones (F_2,27_ = 0.05, p > 0.05).

#### b) Experimental conditions: Composition of turf algal communities and number of species

**Figure 1:**
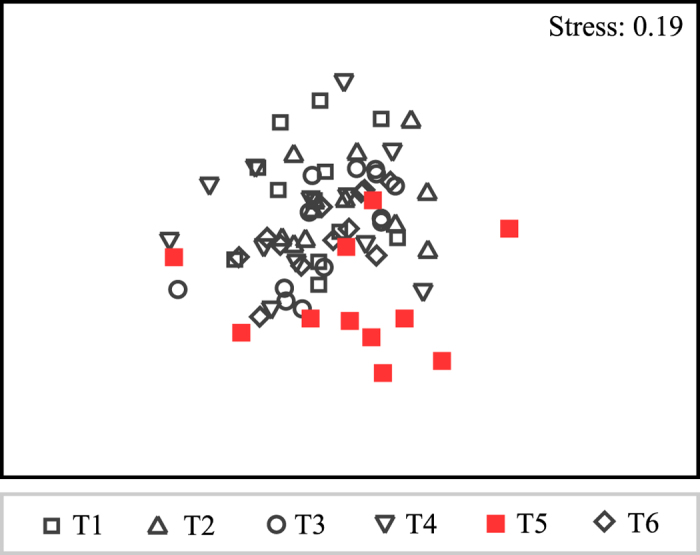
Non-metric multi-dimensional scaling (nMDS) plot of the treatments under experimental conditions. This plot shows the differences in turf algal composition between treatment 5 (T5) and the other treatments (T1–T4, T6). See text for details.

The TA composition differed between treatments (pseudo-F_5,59_ = 1.84, p < 0.05) and dates (pseudo-F_1,59_ = 5.61, p < 0.05), but no interaction between factors was found (pseudo-F_5,59_ = 0.85, p < 0.05); particularly, the composition of TA in contact with coral tissue (Treatment (T) 5) was different from all the other treatments (Fig. 1). According to SIMPER, three taxa (2 Rhodophyta and 1 Cyanobacteria) contributed the most to the differences between treatments: *Centroceras clavulatum* were more frequent, whereas *Dichothrix penicillata* and *Polysiphonia havanensis* were less frequent in T5 than in the other treatments.

The average number of TA species did not differ between the treatments (F_5,70_ = 1.73, p > 0.05) and dates (F_1,70_ = 0.48, p > 0.05), and no interaction between factors was found (F_5,70_ = 0.96, p > 0.05). Algal reproduction was virtually absent. Sexual and asexual reproductive structures were present in only 21 of 1504 specimens analysed (0.46% and 0.93%, respectively); in particular, reproductive structures were absent from the T5 thalli.

### 3) Response of clonal algae under non-manipulated and manipulated conditions

#### a) Non-manipulated conditions: Plasticity and reproduction of *Herposiphonia bipinnata*, *Lophosiphonia cristata,* and *Polysiphonia scopulorum* var. *villum*

**Figure 2:**
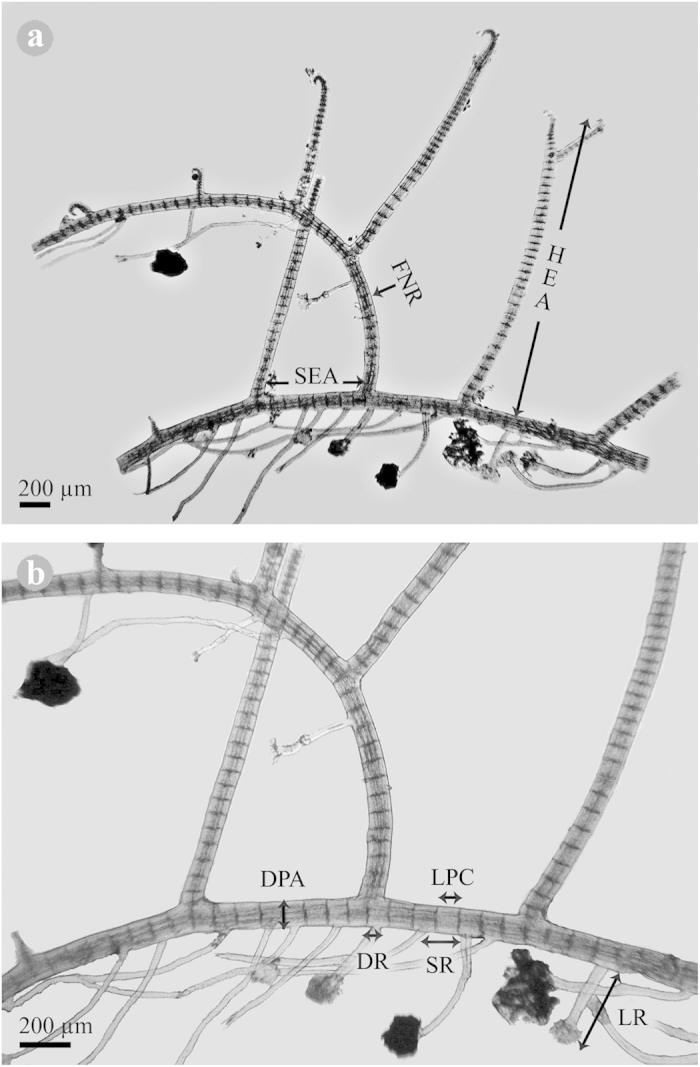
Genet of a ‘typical’ clonal alga, indicating the eight morphological characters evaluated in this study. (**a**) Genet of the red alga *Lophosiphonia cristata*, indicating the distance (“spacer”) between erect axes (SEA), height of the erect axis (HEA), and the formation of new ramets (FNR); (**b**) an enlargement of (a) indicating the diameter of the prostrate axis (DPA), length of pericentral cells of the prostrate axis (LPC), distance (“spacer”) between rhizoids (SR), length of rhizoids (LR), and diameter of rhizoids (DR). Photo credit: H. Bahena-Basave.

The three FTA evaluated under non-manipulated conditions showed differences among the zones (see Supplementary Tables S2 and S3). In the PF, the genets exhibited a shorter SEA and HEA than those from the SF and RE, but a greater DPA, DR, and FNR (Fig. 2, Table 1). The genets observed in both the PF and SF differed from those in the RE in presenting a greater length of the pericentral cells (LPC) of the prostrate axis and length of rhizoids (LR). SR was not affected by the zone in any species (see Supplementary Tables S2 and S3).

The FNR in the three algal species was greater in the PF than in the SF and RE. In *L. cristata*, the FNR was greater in the PF than in SF. In both *L. cristata* and *P. scopulorum* var. *villum*, the LPC was greater in the PF and SF than in the RE. Regarding the other measurements (SEA, HEA, DPA, DR, and LR), only one species (*L. cristata* or *P. scopulorum* var. *villum*) showed differences between the zones (see Supplementary Tables S2 and S3). *Lophosiphonia cristata* exhibited the most characters modified by the zones (SEA, DPA, LPC, DR, FNR), followed by *H. bipinnata* (HEA, LR, FNR) and *Polysiphonia scopulorum* var. *villum* (LPC, FNR).

The presence of reproductive structures was similar in all zones. When the algae were reproducing, the fragments of the three species showed only asexual tetrasporangia.

#### b) Experimental conditions: Plasticity and reproduction of *Polysiphonia scopulorum* var. *villum* and *Parviphycus trinitatensis*

The two clonal species evaluated under the experimental conditions displayed morphological differences between the treatments in direct contact with coral tissue (T1 and T5) and close to it (T6) in comparison with the other treatments (although the differences among treatments was not always well defined) and between dates (see Supplementary Tables S5 and S6). Thus, *P. scopulorum* var. *villum* exhibited a greater distance (“spacer”) between erect axes (SEA), whereas *P. trinitatensis* exhibited more formation of new ramets (FNR) when thalli were in contact with coral tissue than when they were surrounded by TA (Fig. 2, Table 1). In both species, the diameter of the prostrate axis (DPA) was greater when they were in contact with coral tissue than in contact with TA, whereas the height of erect axes (HEA) was similar between the treatments (see Supplementary Tables S5 and S6). In May, both species presented greater FNR, whereas *P. scopulorum* var. *villum* presented a greater SEA and DPA, and *P. trinitatensis* presented a greater HEA and shorter distance (“spacer”) between rhizoids (SR) than in August (see Supplementary Tables S5 and S6). Four significant interactions were registered: for SR and FNR in *P. scopulorum* var. *villum* and for SEA and DPA in *P. trinitatensis* (see Supplementary Tables S5 and S6). Four characters were modified by *P. scopulorum* var. *villum* and two by *P. trinitatensis* between both the different treatments and dates (see Supplementary Tables S5 and S6).

Sexual and asexual reproductive structures developed in *P*. *scopulorum* var. *villum* in 2 of the 14 replicated cores in T3, but not in any of the *P. trinitatensis* plants in T1–T6.

## Discussion

The four stoloniferous clonal algal species in competition for space with *Orbicella* spp. showed morphological changes under both experimental and non-manipulated conditions. These morphological responses have been observed in other clonal algal species when exposed to growth constraints imposed by water movements, substratum, depth, light and nutrient availability, and invasion of new areas (*e.g.*,^12^,^25^,^27^,^28^). The morphological changes of the four clonal algae occurred when they were in contact with or close to the tissue of *Orbicella* spp., in comparison with the genets growing a few centimetres away from the coral−algal boundary. Similarly, morphological responses between the genets of neighbouring algae have been reported when shoots (modules) compete intraspecifically^29^,^30^. However, to our knowledge, this is the first report of morphological responses of clonal algae when they are in competition at the interspecific level.

Five (SEA, HEA, DPA, DR and FNR) of the eight morphological characters evaluated under experimental and non-manipulated conditions were differentially modified among the four FTA species, depending on the coral species involved. For example, *P. scopulorum* var. *villum* increased SEA and DPA when competing with *O. faveolata* but increased FNR when competing with *O. annularis*; the two algal species competing with *O. faveolata* increased DPA, but none of the three species competing with *O. annularis* did; opposite responses were found when *P. scopulorum* var. *villum*, with longer SEA, competed with *O. faveolata*, and when *L. cristata*, with shorter SEA, competed with *O. annularis*. This relative lack of a pattern in the morphological responses of algae in competition with *O. annularis* is similar to the morphological responses observed in clonal vascular plants when searching for resources, where the responses have been shown to differ among species, be absent or be the opposite of the expected responses^17^,^19^,^31^. The FNR exhibited the most regular morphological response, found in the four clonal red algae (but not *P. scopulorum* var. *villum* competing with *O. faveolata*), being greater in genets in contact with coral tissue than in those away from the coral−algal boundary. Similarly, it has been noted that the ramification of the stolon or rhizome of vascular plants (equivalent to the FNR here) is the most consistently modified character within the context of the plasticity of clonal growth forms^19^,^32^.

**Figure 3:**
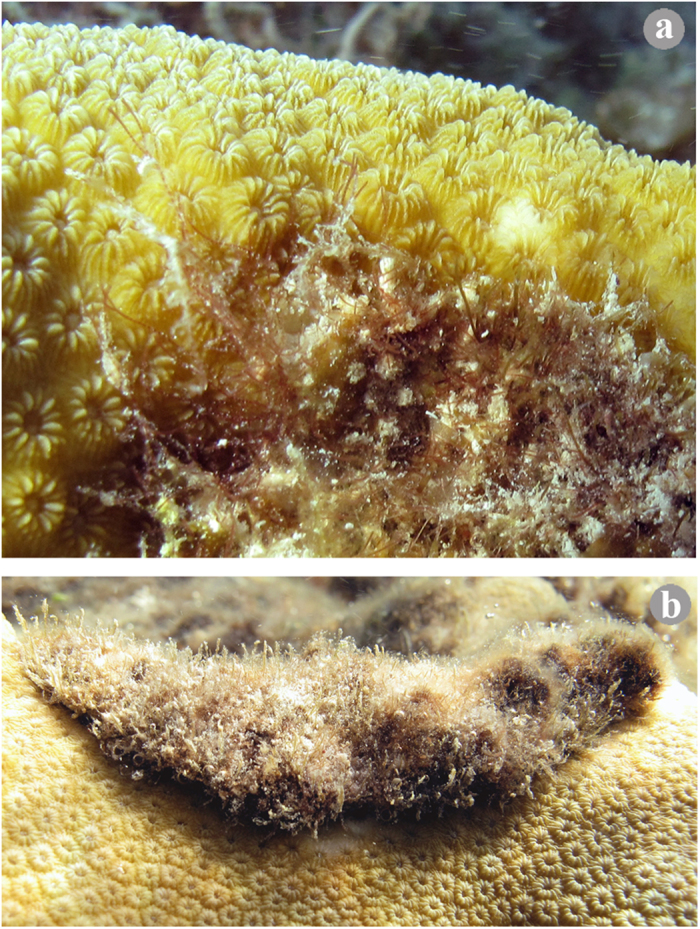
Interactions between the coral *Orbicella annularis* and turf algae containing sediment. (**a**) Filamentous turf algae projecting into coral tissue; note the sediment trapped by some algal axes and in the mat. (**b**) Loose cushions of dense turf algae (with deposited sediment) shading the coral tissue beneath. Photo credit: J. Espinoza-Avalos.

Increased ramification of the rhizomes of vascular plants maximises occupation and dispersion through the substratum^33^,^34^. In addition, a greater number of branches or ramets is a characteristic of plants presenting the phalanx growth form (with short spacers and dense and branched ramets) in comparison with plants presenting the guerrilla growth form (with longer spacers and less spread and branched ramets), and has been proposed as a strategy for excluding competitors and as a better adaptation for stressful conditions, in addition to being useful for resisting the invasion of other plants^15^,^26^,^35^. An increase in the FNR is also observed when algae occur in stressful environmental conditions, such as the charophyte *Chara aspera* exhibiting greater branching under high salinities^36^ and the brown alga *Fucus radicans* exhibiting an increased FNR (adventitious branches) under low salinities^37^. Additionally, the projection of prostrate axes to colonise corals (Fig. 3a), as can occur during the FNR, might indirectly initiate shading of coral tissue by TA^23^ at a microscale due to the formation of loose cushions (Fig. 3b). Based on the above information, the increase in the FNR in the four clonal algae would favour their colonisation of coral tissue as well as resistance to coral invasion and persistence under the stress caused by coral tissue but also a response of TA to the stress caused by corals. Corals might stress algae through their defence and attack mechanisms against competitors, such as the extrusion of mesenteric filaments accompanied by the discharge of nematocysts, swept by tentacles and polyps, growing over the competitor, mucus secretion and allelopathy^5^,^38^,^39^, as partially demonstrated by Nugues *et al.*^40^. In turn, some algae are allopathic to corals^41^. Therefore, both algae and corals exhibit active mechanisms for colonising each other as well as morphological and chemical mechanisms to protect themselves from such colonisation, although the responses between algae and corals depend on the species involved^40^.

We suggest that relatively few algal species are well adapted to remain next to coral tissue and compete for space with *Orbicella* spp.; other algae show better performance in microenvironments away from contact with coral. This suggestion is based on the lower number of both total species and exclusive species registered in the PF compared to those found in the other zones under non-manipulated conditions, and the differential composition of algal species in contact with or close to coral tissue *vs.* those not in contact with coral under both experimental and non-manipulated conditions. Nevertheless, the relatively low number of species in the PF represents a relatively high richness within a small band (0.5 cm) next to coral tissue, with many species presenting a clonal growth pattern. According to the clonal growth types differentiated by Collado-Vides^25^ and the thallus growth forms indicated through descriptions, diagrams, and photographs by several authors (*e.g.*,^42^,^43^,^44^,^45^), we conservatively considered that 38 of the 53 species found in the PF under non-manipulated conditions and that 38 of the 65 species found in T1, T5, and T6 under experimental conditions were clonal algae. Of the clonal species registered under both conditions, 22 presented the same clonal growth type (stoloniferous) as the four algae in which we assessed morphological plasticity (see Supplementary Table S1). It is likely that some of those 22 stoloniferous clonal algae also present a diversity of responses in their morphological characters, including a greater FNR.

Turf algal assemblages with a relatively high richness, including groups of clonal algae with matching morphologies and phenotypic responses (showing functional redundancy, especially in the FNR), can promote the resilience of TA assemblages that compete with *Orbicella*, hindering the coral from re-occupying the space lost by the corals or gained by TA, due to complementarity in the competitive skills of clonal algae. Similarly, relatively diverse communities of vascular plants resist or reduce the successful invasion of exotic species^46^,^47^,^48^, and among seaweeds, the presence of different assemblages leads to resistance of invasion by the brown alga *Sargassum muticum*^49^. Additionally, sessile marine invertebrate communities with a richness of as low as four species can reduce the success of the invasion of space by competitors^50^. Naeem *et al.*^51^ observed a reduction in the success of invasive plants when using polycrops of up to 25 species, which is a low number in comparison with the species richness (ranging from 53 to 65) of the algal assemblages found in the vicinity of *Orbicella* tissue. Functional redundancy, or the capacity of a species to functionally compensate for the loss of another species with the same geometry and to occupy the same space or to be located at a short distance^52^,^53^,^54^, is important for maintaining resiliency^52^ and represents a form of biological insurance against the loss of any other redundant species^50^. For example, some plankton communities are resilient to disturbances due to the replacement of species that show similar performance in ecological functions^55^. Hence, the functional redundancy of clonal TA would allow them to be resilient when growing close to coral tissue, resisting potential attacks from the coral as a group, while the TA would simultaneously continue to exert their stressing effects on *Orbicella* spp.^23^,^24^.

In summary, algal assemblages responded at the species and community levels to competition for space with the coral *Orbicella* spp., and the responses of the algae in contact with or close to the coral tissue were significantly different from those growing a few centimetres away from the coral−algal boundary. The TA growing next to the tissue of *Orbicella* spp. displayed high responses in morphological characters of their genets as well as the same growth form (phalanx), relatively high species richness, and species with functional redundancy. We suggest that all of these specific and community responses favour the colonisation of coral tissue by the algae, allowing resistance to invasion by the coral and persistence of algal assemblages under the stress caused by coral tissue, thus providing stability to algal assemblages competing with coral and hindering corals from re-occupying the space lost by the corals or gained by TA. The different composition of the algal assemblage of the PF, generally involving fewer total species and few exclusive species, suggests that species of TA are not homogeneously adapted to withstand potential attacks from coral and to compete successfully for space with *Orbicella* spp. Our results also suggest that fundamental active interactions between TA and *Orbicella* spp. occur over a distance of less than 1 cm between the two types of organisms and that a few centimetres away from that interface, the coral skeleton may simply serve as another hard substrate that can be colonised by additional species of algae that are not necessarily adapted to compete with corals. The high abundance of clonal algae bordering coral tissue gives them an additional competitive advantage because when ramets are lost through physical disturbances or herbivory, unaffected genets will rapidly re-occupy the space through vegetative propagation^56^. In addition, remnants or prostrate axes left behind after disturbances will regenerate erect axes^8^; for example, some TA species surrounding *O. annularis* regenerate erect axes from scarcely visible remnants of prostate axes left after the algae are manually scraped from the coral skeleton. To our knowledge, this is the first assessment of the morphological and community responses of TA in competition for space with corals. Our results suggest that these responses may favour the colonisation of coral tissue and the permanence of TA mats around the periphery of the coral. This small-scale study in the coral−TA interaction boundary revealed important, previously unnoticed information.

## Methods

### Study area

**Figure 4:**
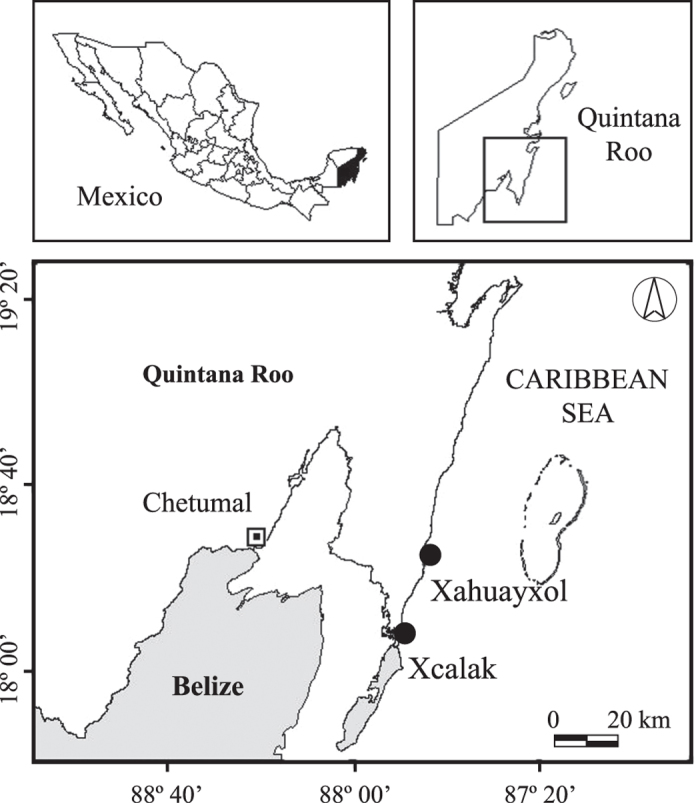
Study sites in the southern part of Quintana Roo, Mexico. The study sites are located in reef lagoon environments at Xcalak and Xahuayxol (black circles). This figure was made using GIS software (ArcView 3.3) and Adobe Illustrator CS4.

The study was carried out at Xcalak (18°15′41.6”N, 87°49′30”W) and Xahuayxol (18°30′11.9”N, 87°45′24.8”W), in the southern part of Quintana Roo, Mexican Caribbean (Fig. 4). Both sites are located in reef lagoons (approximately 1.5 and 2 m deep, respectively) near the breaker zone, within a Marine Protected Area (National Park “Arrecifes de Xcalak”). The reef lagoon at both study sites has a predominantly sandy bottom with seagrass beds of *Thalassia testudinum* and *Syringodium filiforme*, macroalgae, Alcyonacea corals, and patches or aggregations of stony corals^57^. The Xcalak and Xahuayxol fringing reefs have similar average annual sea surface temperatures and salinities (27.80 °C and 27.81 ºC, and 35.79 and 35.78, respectively see^58^), low coral cover (8.7% and 11.7%, respectively^59^) in fore reefs (~10 m), and high algal cover (41%^60^ and 58%^61^, respectively). *Orbicella* is the most abundant coral genus (50% of relative abundance), followed by *Diploria* (12%)*, Siderastrea* (11%)*, Porites* (9%)*, Agaricia* (5%)*, Montastraea* (4%)*, Colpophyllia* (4%) and *Acropora* (3%)^58^,^62^. The coral species used for the experimental conditions (in Xcalak) and non-manipulated conditions (in Xahuayxol) were selected to better accomplish each objective of the study. At Xcalak, the morphological shape of the abundant coral *Orbicella faveolata* (frequently with relatively flat surfaces and large size, 2–3 m in diameter) facilitated the extraction of coral cores and to the reciprocal transplantations of live and dead (covered by TA) coral cores. At Xahuayxol, the dominant coral *O. annularis*, with individual lobules or ramets interacting with TA in a short perimeter of coral tissue (diameter ≥ 14 cm^63^), allowed us to collect TA from the whole periphery of ramets. The relative abundance of benthic algae at the Xcalak and Xahuayxol fore reefs was highest for turf algae (51% and 53%, respectively) in comparison to coralline algae and macroalgae (32% and 29%, and 16% and 18%, respectively^59^). The assemblages of TA at Xahuayxol were approximately 8 mm in height and included abundant sediment with grains of less than 0.3 mm. The assemblages at both sites were mainly composed of *Polysiphonia* spp., *Lophosiphonia cristata*, *Parviphycus trinitatensis*, *Herposiphonia* spp., *Centroceras clavulatum*, *Amphiroa fragilissima*, *Jania* spp., *Ceramium* spp., *Padina* sp., *Lyngbya* spp. and *Dichothrix* spp., while *Bryobesia johannae* and *Anotrichium tenue* were common at Xahuayxol but not at Xcalak, and *Sphacelaria* sp. was common at Xcalak but not at Xahuayxol^24^,^64^.

### Sampling design and methods

#### Experimental conditions

##### Coral cores used as transplants and controls.

**Figure 5:**
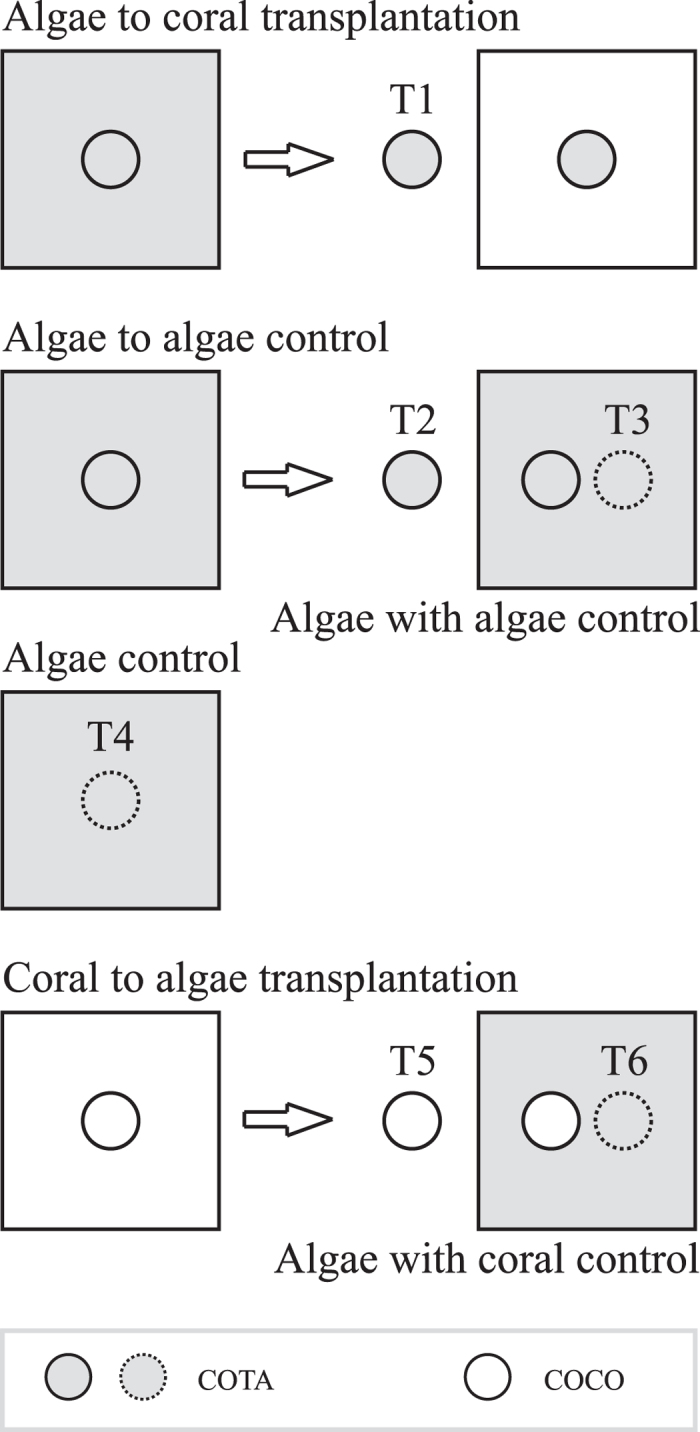
Graphic representation of the six treatments carried out on the coral *Orbicella faveolata* under the experimental conditions. The experimental design includes healthy ( 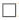 ) and dead coral colonies ( 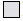), healthy (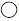) and dead (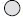) transplanted coral cores covered by turf algae (TA), and control cores (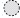). Treatments: T1 = Algae to coral transplant; T2, T3, and T4 = control for T1; T5 = coral to algae transplant; and T6 = control for T5. COCO = cores covered with coral tissue; COTA = coral skeleton cores covered with TA. See text for details.

In order to evaluate the potential responses i) in morphological plasticity of the most abundant clonal filamentous algae (*Polysiphonia scopulorum* var. *villum* and *Parviphycus trinitatensis*) and ii) in the composition of turf algal (TA) communities towards the presence of the coral *O. faveolata* (*e.g.*, competition for space), two different types of coral cores were reciprocally transplanted: i) cores covered with coral tissue (COCO), and ii) coral skeleton cores covered with TA (COTA). Both were transplanted to hosting coral colonies; controls were COTA left intact during the experiment (Fig. 5). The cores measured 5 cm in diameter (16.8 cm^2^) and approximately 2 cm in depth and were obtained with a pneumatic drill. The cores were cemented with marine epoxy in a hole previously made with the pneumatic drill in the coral colony hosting the transplants. Each core was identified with a steel rectangle (15 × 55 mm, marked with letters and numbers) nailed to a dead portion of the hosting colony. The top part of the implanted cores and the external surface of the hosting colony were accommodated at a similar level see^23^.

##### Algae to coral transplantation.

COTA growing on dead corals were transplanted into healthy *O. faveolata* colonies (treatment 1 (T1) in Fig. 5). Three controls were carried out for these transplants. The first consisted of COTA growing on dead corals transplanted into dead corals covered with algae (Fig. 5, T2), to test whether the biological parameters and species composition of TA were affected by the transplant manipulation. The second control consisted of TA growing on dead corals collected next to the cores used as the first control (Fig. 5, T3), to test whether algae covering dead coral colonies were affected by receiving the transplanted coral cores. The third control consisted of COTA obtained from non-manipulated TA growing on dead corals (Fig. 5, T4).

##### Coral to algae transplantation.

COCO were transplanted into dead coral colonies covered by TA (Fig. 5, T5). The controls for this treatment consisted of TA growing on dead coral collected next to the coral transplants (Fig. 5, T6). They were used to test whether TA growing on dead coral were affected by receiving the healthy COCO.

Before initiating the experiment, we placed 42 cores to be used in T1, T2, and T5 (n = 7 cores per treatment and date) inside the upper releasable part of two-part PVC tubes. The lower part of the tubes was cemented into a 50 × 42 × 10 cm concrete block. The cores in the tubes were left next to the experimental coral colonies for 3 weeks to allow regeneration of coral and algal tissue at their damaged edges. At the end of the regeneration time, 36 cores (n = 6 cores per treatment and date) were selected for the experiment, based on the best-cut skeleton cores and the presence of live coral or algae in all the periphery of cores.

The T1, T2 and T5 cores were placed under the experimental conditions in November 2003 (month 0), and cores from all treatments were extracted in May (month 6) and August 2004 (month 9). All the extracted cores were preserved in 4% formaldehyde in seawater.

#### Non-manipulated conditions

On 25 May 2010, TA that had overgrown *O. annularis* coral tissue ramets (the lobes of a coral colony) were collected at Xahuayxol from three zones at different distances from the *O. annularis*−algal boundary: from the first 0.5 cm next to *O. annularis* tissue (primary front, PF); from a 1-cm-wide band located 3–4 cm from the coral tissue (secondary front, SF); and from ≥30 cm away from the periphery of coral tissue (rearguard zone, RE). The TA area sampled was similar in all three zones. Ten samples per zone were collected with the aid of a knife and preserved in vials with 4% formaldehyde in seawater.

#### Experimental and non-manipulated conditions

##### Composition of turf algal communities, number of species, and percentage of species undergoing reproduction

. The species composition and number of species were recorded after all the individuals/ramets of algae in each sample from both the experimental (cores of 16.8 cm^2^) and non-manipulated (samples of 4 cm^2^) conditions had been observed and identified. TA were identified, usually to species level, using common keys^42^,^43^,^44^,^45^ (with updated nomenclature from Algaebase^65^) and stereoscopic (magnifications: 0.67–4.5X) and compound (magnifications: 5X, 10X, 40X and 100X) Olympus microscopes. Diverse techniques were employed to identify the algal taxa depending on the specimen (e.g., decalcification, staining, transversal and longitudinal cuts, observations of cell shape and arrangement, cell size measurements (long and wide), and counting the number of pericentral cells and cell layers). Reproductive data were obtained only for the algae from the non-manipulated samples as follows: i) the percentage of species presenting sexual/asexual reproductive structures was obtained by examining all individuals/ramets in each sample; and ii) the presence of sexual/asexual reproductive structures in each of the species *Herposiphonia bipinnata*, *Lophosiphonia cristata* and *Polysiphonia scopulorum* var. *villum* was obtained by examining ramets in each sample. Overall, 113 slides of 84 algal taxa from the experimental conditions and 180 vials of 90 algal taxa from the non-manipulated conditions were deposited at the CIQR Herbarium at ECOSUR.

##### Plasticity and reproduction of *Herposiphonia bipinnata*, *Lophosiphonia cristata*, *Polysiphonia scopulorum* var. *villum* (Ceramiales), and *Parviphycus trinitatensis* (Gelidiales)

. Four clonal (*i.e.*, algae with fragmented parts of the thallus, ramets, having the potential to reattach to the substratum and continue growing as independent organisms^25^) Rhodophyta species from two orders (Ceramiales and Gelidiales) growing in contact with the *Orbicella* spp. tissue were selected for the study, based on their high frequency in the pre-analysed samples and their same growth form (*i.e.*, filamentous stoloniferous algae with prostrate axes from which erect axes emerge). *Polysiphonia scopulorum* var. *villum* and *P. trinitatensis* in contact with *O. faveolata* were selected to evaluate morphological characters under the experimental conditions. Similarly, *Herposiphonia bipinnata*, *L. cristata,* and *P. scopulorum* var. *villum* in contact with *O. annularis* were selected to evaluate the morphology responses and reproduction under the non-manipulated conditions.

In order to evaluate the potential effect of the coral−algal competition on the responses in morphological plasticity of clonal algae, eight morphological characters were evaluated (Fig. 2 and Table 1). Five morphological characters (a−c, e and h) were recorded for *P. scopulorum* var. *villum* and *P*. *trinitatensis* under the experimental conditions. The results from the experimental part of the study and the experience gained working with small clonal algae allowed us to evaluate eight morphological characters (a−h) for *H. bipinnata*, *L. cristata,* and *P. scopulorum* var. *villum* (Ceramiales) under the non-experimental conditions.

Under the experimental conditions, six fragments per species in each core (per treatment and date) were selected to evaluate the five morphological characters. A total of 12 average readings (6 samples per treatment and date) were obtained for *P. scopulorum* var. *villum* and *P. trinitatensis* for each morphological character. Under the non-manipulated conditions, five fragments per species from each sample per zone were selected to evaluate all morphological characters. A total of 24 average readings (8 samples per zone) were obtained for *H. bipinnata* and *P. scopulorum* var. *villum*, and 30 average readings (10 samples per zone) were obtained for *L. cristata* for each morphological character. The first two species were absent in two of ten samples from two zones; therefore, the number of samples analysed was equalised to 8. Thirty samples (10 samples per zone) were examined to register the presence/absence of reproductive structures for each species.

##### Statistical analyses.

Shapiro-Wilk and Levene tests were used to determine normality and homogeneity of variances of the average values of the number of species, the percentage of species with reproductive structures in the TA samples, and the morphological characters of the four clonal species. One- and two-way analyses of variance (ANOVA) were performed on the data obtained under the non-manipulated conditions (factor: zone; untransformed data) and experimental conditions (factors: treatment and extraction date; square root-transformed data), respectively. Scheffe and Games-Howell *post hoc* tests were subsequently applied to the data for the non-manipulated conditions (the last test, only for data without homoscedasticity); Student Newman-Keuls (SNK) *post hoc* tests were performed for the experimental conditions. The data (presence/absence of species) from the TA samples were analysed by one- and two-way permutational multivariate analysis of variance (PERMANOVA) to evaluate the difference in the specific composition between zones (non-manipulated conditions) and between treatments and dates (experimental conditions), respectively. Analyses were performed based on a Bray-Curtis matrix using Type III (partial) sums of squares and unrestricted permutation of raw data with 999 permutations. A non-metric multi-dimensional (nMDS) plot, using Bray-Curtis similarity, was performed to show the spatial distribution of algal composition in the treatments under experimental conditions. Subsequently, SIMPER analyses were performed to determine which turf species contributed to the differences observed in both non-manipulated and experimental conditions. To evaluate whether the presence/absence of reproductive structures in *H. bipinnata*, *L. cristata* and *P. scopulorum* var. *villum* depended on the zone of collection in the non-manipulated conditions, Pearson chi-square tests were applied.

## Additional Information

**How to cite this article**: Cetz-Navarro, N. P. *et al.* Morphological and community changes of turf algae in competition with corals. *Sci. Rep.* **5**, 12814; doi: 10.1038/srep12814 (2015).

## Supplementary Material

Supplementary Information

## Figures and Tables

**Table 1:** Morphological measurements (a.–g. in μm) conducted in Herposiphonia bipinnata*, *Lophosiphonia cristata*, *Parviphycus trinitatensis* and *Polysiphonia scopulorum* var. *villum* growing on *Orbicella* spp. under non-manipulated (N) and experimental (E) conditions.

**Morphological characters**		**Abbreviation**	**Condition**
a.	Distance (“spacer”) between erect axes	SEA	N, E
b.	Height of erect axis	HEA	N, E
c.	Diameter of prostrate axis	DPA	N, E
d.	Length of pericentral cells of the prostrate axis (*i.e.*, distance between two upright axes, measured from the centre to the centre of axes, divided by the number of segments of pericentral cells found between the axes)	LPC	N
e.	Distance (“spacer”) between rhizoids	SR	N, E
f.	Length of rhizoids	LR	N
g.	Diameter of rhizoids (measured in close proximity to the prostrate axis)	DR	N
h.	Percentage of fragments with formation of new ramets (*i.e.*, a branch of undetermined growth - with new erect axes, stolon and rhizoids - formed by vegetative growth from an otherwise determined erect axis of the genet)	FNR	N, E

See text and Fig. 2 for details.

*The formation of erect axes or branches in *H. bipinnata* is different from the other three species because *H. bipinnata* produces pairs of erect branches, one of determinate growth and another that can be of indeterminate growth or truncated (cut-off branch), separated by four segments of pericentral cells. Consequently, in *H. bipinnata*, the percentage of FNR was obtained as the ratio of indeterminate/cut-off branches, and the spacer of erect axes was measured between erect determined axes.
