# Supplementary material for: Morphological and community changes of turf algae in competition with corals
Source: Sci Rep. 2015 Aug 5;5:12814. doi: 10.1038/srep12814 (PMC4525492; doi:10.1038/srep12814)
Supplement: Supplementary Information [file srep12814-s1.pdf]

Morphological and community changes of turf algae in competition with corals

Neidy P. Cetz-Navarro, Lizette I. Quan-Young & Julio Espinoza-Avalos

Content

Results for turf algae (TA) under non-manipulated conditions (zones)

Supplementary Table S1 …… 1

Supplementary Table S2 …… 6

Supplementary Table S3 …… 8

Results for turf algae (TA) under the experimental conditions (treatments and dates)

Supplementary Table S4 …… 12

Supplementary Table S5 …… 16

Supplementary Table S6 …… 18

Results for turf algae (TA) under non-manipulated conditions (zones)

Supplementary Table S1 | Presence (1) and absence (0) of taxa of turf algae (TA; algae and cyanobacteria) living in three zones in relation to *Orbicella annularis* under non-manipulated conditions: primary front (FP, in contact with coral tissue); secondary front (FS, 3-4 cm from the tissue border); and rearguard (RE, ≥ 30 cm away from coral tissue, overgrowing dead corals). The presence of

reproductive structures in the thalli of the TA in each zone is represented with an asterisk. 1 indicates an exclusive presence of the species in the marked zone. For clonal species, the type of clonal growth (CG) is included: Fr. Fragmentation of trichome; St, stoloniferous; Re, branching bending and re-attachment; Ho, holdfast

**Taxa**	**PF**	**SF**	**RE**	**CG**
**Cyanobacteria**				
Dichothrix fucicola	0	0	1	Fr
Dichothrix penicillata	1	1	1	Fr
Dichothrix utahensis	1	1	1	Fr
Lyngbya confervoides	1	1	1	Fr
Lyngbya majuscula	0	0	1	Fr
Lyngbya sordida	1	1	1	Fr
Rivularia sp.	0	0	1	Fr
Spirulina sp.	1	1	1	Fr
Cyanobacteria 1	1	1	1	Fr
Cyanobacteria 2	1	0	0	Fr
Cyanobacteria 3	0	1	1	Fr
Cyanobacteria 4	1	1	1	Fr
Cyanobacteria 5	0	0	1	Fr
Subtotal (13 taxa)	8	8	12	
**Rhodophyta**				
Amphiroa fragilissima	0	1	1	Ho
Anotrichium tenue	1*	1	1	St
Centroceras clavulatum	1*	1*	1*	Re
Ceramium brevizonatum var. *caraibicum*	0	1	1*	St
Ceramium cimbricum	1*	1*	1*	St
Ceramium cimbricum f. *flaccidum*	1	1	0	St
Ceramium cruciatum	1	1	0	St
Ceramium sp.	1*	1*	1*	St
Ceratodictyon variabile	1	0	1	St
Champia parvula var. *prostrata*	0	1*	1	Re
Champia vieillardii	0	0	1	Re
Chondria baileyana	1	1	1	

Chondria cnicophylla	1	1	1*	Ho
Chondria collinsiana	1	1*	1*	
Chondria floridana	0	1	1	
Chondria leptacremon	1	1	1*	Ho
Chondria sp.	1	0	0	
Corallophila verongiae	1	1	1*	St
Crouania mayae	1	0	1	
Dasya corymbifera	0	1	0	
Gayliella flaccida	1	1*	1*	St
Gelidiopsis sp.	0	0	1	St
Griffithsia globulifera	0	1	1	
Herposiphonia bipinnata	1*	1*	1*	St
Herposiphonia pecten-veneris var. *laxa*	1	1	1	St
Herposiphonia secunda f. *tenella*	0	0	1	St
Hypnea spinella	0	1	1	Re
Jania adhaerens	0	1	1	Re
Jania capillacea	1	1	1	Ho, Re
Jania pumila	0	1	1	Ho
Laurencia cervicornis	0	1	1	Ho
Laurencia sp.	1	0	1	
Lophosiphonia cristata	1*	1*	1*	St
Neosiphonia ferulacea	1*	1*	1*	St
Neosiphonia gorgoniae	1*	1*	1*	Re
Neosiphonia sertularioides	1	1	1	St
Neosiphonia sphaerocarpa	1*	1*	0	St
Neosiphonia sp.	0	1	0	
Palisada perforata	0	1	0	Ho
Parviphycus setaceus	1	0	1	St, Re
Parviphycus trinitatensis	1*	1*	1*	St, Re
Polysiphonia binneyi	1*	1*	0	
Polysiphonia howei	0	1	1	St
Polysiphonia pseudovillum	1	1*	0	St
Polysiphonia scopulorum	1	1	1	St
Polysiphonia scopulorum var. *villum*	1*	1*	1*	St
Polysiphonia sp.	1*	0	0	
Pterocladiella sanctarum	0	1	0	St, Re
Stylonema alsidii	1	1	1	

Taenioma nanum	1*	1*	1*	St
Yuzurua poiteaui	1	1	1	
Yuzurua poiteaui var. *gemmifera*	0	1	1	Ho
Ceramiaceae 1	1	1	1	
Ceramiaceae 2	0	0	1	
Rhodophyte 1	0	1	0	Ho
Subtotal (55 taxa)	35	45	41	
**Chlorophyta**				
Anadyomene sp.	0	0	1	
Boodleopsis pusilla	0	0	1	St
Boodleopsis verticillata	0	0	1	
Bryobesia johannae	1	1	1	
Bryopsis ramulosa	1	0	0	St
Cladophora laetevirens	0	0	1	
Cladophora liniformis	1	1	1	
Cladophora vagabunda	0	1	1	St
Cladophora sp.	1	0	0	
Cladophoropsis membranacea	0	0	1	St, Re
Derbesia fastigiata	1	1	1	Re
Derbesia turbinata	0	1	0	Re
Halimeda sp.	0	1	1	Fr
Parvocaulis polyphysoides	0	0	1	
Parvocaulis pusillus	0	0	1	
Siphonocladus rigidus	1	1*	1*	Re
Ulva flexuosa subsp. *paradoxa*	1	1	1	
Chlorophyte 1	0	0	1	
Subtotal (18 taxa)	7	8	15	
**Phaeophyceae**				
Canistrocarpus cervicornis	0	0	1	Re
Dictyerpa stage of *Padina*	1	1	1	
Dictyota caribaea	0	1	0	Re
Dictyota sp.	0	1	1	Fr
Lobophora variegata	1	1	1	Re
Padina gymnospora	0	1	1	
Sargassum sp.	0	0	1	
Sphacelaria novae-hollandiae	1*	0	0	St

Sphacelaria tribuloides	0	1*	0	St
Sphacelaria sp.	0	0	1	
Subtotal (10 taxa)	3	6	7	
TOTAL (96 taxa)	53	67	75	

Supplementary Table S2 | Mean values (± standard deviation) of the morphological characters of *Herposiphonia bipinnata*, *Lophosiphonia cristata* and *Polysiphonia scopulorum* var. *villum* growing in the primary (PF), secondary front (SF) and rearguard (RE) zones in relation to *Orbicella annularis* under non-manipulated conditions

Morphological characters	*Herposiphonia bipinnata*	*Lophosiphonia cristata*	*Polysiphonia scopulorum* var. *villum*
**Distance between erect axes (µm)**			
PF	288.6 (± 70.3)	359.8 (± 66.5)	293.9 (± 55.8)
SF	278.0 (± 31.5)	500.8 (± 78.9)	364.3 (± 47.1)
RE	281.6 (± 38.8)	495.2 (± 78.4)	330.6 (± 79.4)
**Height of erect axis (µm)**			
PF	2401 (± 601)	3470 (± 593)	1342 (± 173)
SF	3345 (± 1374)	4257 (± 1070)	1713 (± 289)
RE	3030 (± 636)	3441 (± 1417)	1558 (± 130)
**Diameter of prostrate axis (µm)**			
PF	53.7 (± 6.8)	94.4 (± 7.2)	93.5 (± 12.0)
SF	53.0 (± 2.5)	86.3 (± 10.1)	96.1 (± 7.9)
RE	52.1 (± 7.2)	80.4 (± 4.4)	96.4 (± 6.7)
**Length of pericentral cells of the prostrate axis (µm)**			
PF	70.4 (± 5.6)	82.5 (± 7.4)	120.7 (± 15.1)
SF	68.2 (± 5.4)	77.1 (± 8.5)	108.2 (± 7.5)
RE	58.8 (± 8.7)	65.6 (± 5.4)	106.5 (± 11.9)
**Distance between rhizoids (µm)**			
PF	288.1 (± 33.6)	207.5 (± 26.0)	310.8 (± 106.5
SF	318.1 (± 54.9)	218.4 (± 44.4)	316.2 (± 54.5)
RE	304.3 (± 39.4)	217.6 (± 26.9)	300.1 (± 103.0)
**Length of rhizoids (µm)**			
PF	478.3 (± 128.7)	578.8 (± 103.5)	437.0 (± 38.1)
SF	425.9 (± 47.6)	510.8 (± 99.5)	432.5 (± 30.9)
RE	422.3 (± 124.6)	481.2 (± 72.5)	335.8(± 53.0)
**Diameter of rhizoids (µm)**			
PF	38.8 (± 4.5)	40.0 (± 2.5)	38.6 (± 5.5)

SF	39.5 (± 4.0)	34.0 (± 2.7)	37.1 (± 3.2)
RE	39.8 (± 5.5)	33.4 (± 1.4)	39.5 (± 1.9)
**Formation of new ramets (%)**			
PF	33.9 (± 9.3)	55.1 (± 6.5)	71.2 (± 11.0)
SF	22.1 (± 7.1)	26.2 (± 6.8)	53.5 (± 12.8)
RE	13.3 (± 7.0)	17.7 (± 4.2)	58.6 (± 4.7)

Supplementary Table S3 | Results of one-way ANOVA of the effects on the morphological characters of *Herposiphonia bipinnata*, *Lophosiphonia cristata* and *Polysiphonia scopulorum* var. *villum* growing in the primary front (FP), secondary front (FS) and rearguard (RE) zones of *Orbicella annularis* under non-manipulated condition. ns = not significant

**Morphological characters**	**SS**	**df**	**MS**	**F**	**p**	**Conclusion**
**Distance between erect axes**						
Herposiphonia bipinnata						
Between groups	466.53	2	2334.26	0.10	0.911	ns
Within groups	52027.95	21	2477.52			
Total	52494.47	23				
Lophosiphonia cristata						
Between groups	127578.34	2	63789.17	11.40	0.000	PF < SF, RE
Within groups	151134.68	27	5597.59			
Total	278713.02	29				
Polysiphonia scopulorum var. *villum*						
Between groups	19843.79	2	49921.90	2.56	0.101	ns
Within groups	81530.36	21	3882.40			
Total	101374.14	23				
**Height of erect axis**						
Herposiphonia bipinnata	556106.25	2	278053.13	6.40	0.007	PF < SF, RE
Between groups	912793.75	21	43466.40			
Within groups	1468900.00	23				
Total						
Lophosiphonia cristata						
Between groups	4289943.22	2	2144971.61	1.84	0.179	ns
Within groups	31546996.60	27	1168407.28			
Total	35836939.80	29				
Polysiphonia scopulorum var. *villum*						
Between groups	3702093.58	2	1851046.79	2.09	0.148	ns
Within groups	18577301.75	21	884633.42			
Total	22279395.33	23				
**Diameter of prostrate axis**						
Herposiphonia bipinnata						

Between groups	41.65	2	20.82	0.25	0.782	ns
Within groups	1756.59	21	83.65			
Total	1798.24	23				
Lophosiphonia cristata						
Between groups	988.82	2	494.41	8.52	0.001	PF > RE
Within groups	1565.93	27	58.00			
Total	2554.75	29				
Polysiphonia scopulorum var. *villum*						
Between groups	10.44	2	5.22	0.15	0.862	ns
Within groups	735.57	21	35.03			
Total	746.01	23				
**Length of pericentral cells of the prostrate axis**						
Herposiphonia bipinnata						
Between groups	969.60	2	484.80	3.41	0.052	ns
Within groups	2988.36	21	142.30			
Total	3957.96	23				
Lophosiphonia cristata						
Between groups	1488.86	2	744.43	14.32	0.000	PF, SF > RE
Within groups	1403.30	27	51.97			
Total	2892.16	29				
Polysiphonia scopulorum var. *villum*						
Between groups	606.44	2	303.22	6.70	0.006	PF, SF > RE
Within groups	951.33	21	45.30			
Total	1557.77	23				
**Distance between rhizoids**						
Herposiphonia bipinnata						
Between groups	3593.21	2	1796.61	0.95	0.404	ns
Within groups	22233.40	21	1058.73			
Total	26284.43	23				
Lophosiphonia cristata						
Between groups	737.13	2	368.56	0.33	0.723	ns
Within groups	30331.39	27	1123.38			
Total	31068.52	29				
Polysiphonia scopulorum var. *villum*						
Between groups	1061.39	2	530.69	0.06	0.938	ns
Within groups	174369.56	21	8303.31			

Total	175430.95	23				
**Length of rhizoids**						
Herposiphonia bipinnata						
Between groups	52353.00	2	26176.50	15.07	0.000	PF, SF > RE
Within groups	36481.50	21	1737.21			
Total	88834.50	23				
Lophosiphonia cristata						
Between groups	50086.40	2	25043.2	2.90	0.072	ns
Within groups	232884.80	27	8625.36			
Total	282971.20	29				
Polysiphonia scopulorum var. *villum*						
Between groups	15712.75	2	7856.38	0.69	0.514	ns
Within groups	240523.88	21	11453.52			
Total	256236.63	23				
**Diameter of rhizoids**						
Herposiphonia bipinnata						
Between groups	23.04	2	11.52	0.78	0.473	ns
Within groups	311.58	21	14.84			
Total	334.62	23				
Lophosiphonia cristata						
Between groups	264.02	2	132.01	25.31	0.000	PF > SF, RE
Within groups	140.85	27	5.22			
Total	404.87	29				
Polysiphonia scopulorum var. *villum*						
Between groups	3.87	2	1.93	0.09	0.917	ns
Within groups	467.41	21	22.26			
Total	471.28	23				
**Formation of new ramets**						
Herposiphonia bipinnata						
Between groups	0.13	2	0.07	6.34	0.007	PF > SF, RE
Within groups	0.22	21	0.01			
Total	0.35	23				
Lophosiphonia cristata						
Between groups	7688.11	2	3844.05	108.15	0.000	PF > SF > RE
Within groups	959.70	27	33.55			
Total	8647.81	29				

Polysiphonia scopulorum var. *villum*						
Between groups	0.18	2	0.09	14.28	0.000	PF > SF, RE
Within groups	0.13	21	0.01			
Total	0.30	23				

Results for turf algae (TA) under the experimental conditions (treatments and dates)

Supplementary Table S4 | Presence (1) and absence (0) of taxa of turf algae (TA; algae and cyanobacteria) growing in the experimental core treatments (T1-T6) in *Orbicella faveolata* under the experimental conditions in May and August. Treatments: T1= Algae to coral transplantation; T2, T3, and T4= control for T1; T5= coral to algae transplant; and T6= control for T5 (see Fig. 1 for details). The presence of reproductive structures in the taxa of TA in each treatment is represented with an asterisk. For clonal species, the type of clonal growth (CG) is included: Fr, fragmentation of trichome; St, stoloniferous; Re, branching bending and re-attachment; Ho, holdfast

**Taxa**	**May**						**August**						
	**T1**	**T2**	**T3**	**T4**	**T5**	**T6**	**T1**	**T2**	**T3**	**T4**	**T5**	**T6**	**CG**
**Cyanobacteria**													
Dichothrix penicillata	0	0	1	0	1	1	0	0	0	0	1	0	Fr
Dichothrix utahensis	0	0	1	0	1	1	0	0	0	0	1	0	Fr
Lyngbya confervoides	1	1	1	1	1	1	0	1	1	1	0	0	Fr
Lyngbya majuscula	0	0	1	1	1	1	1	1	0	1	1	1	Fr
Lyngbya sordida	1	1	1	1	1	1	1	0	1	1	0	1	Fr
Symploca sp.	1	0	0	0	0	1	0	0	0	0	0	0	Fr
Subtotal (6 taxa)	4	3	5	4	5	6	3	3	3	4	3	3	
**Rhodophyta**													
Amphiroa fragilissima	1	1	1	1	1	1	0	1	1	1	0	1	Ho
Anotrichium tenue	0	0	0	1	0	0	1	0	1	0	1	1	St
Anotrichium sp.	0	0	1	0	0	0	0	0	0	0	0	0	
Asparagopsis taxiformis “Falkenbergia" stage	0	0	0	0	1	0	0	0	0	0	0	0	
Bostrychya sp.	0	0	0	0	0	0	1	1	1	1	1	1	
Centroceras clavulatum	1	1	1	1	1	1	1	1	1	1	1	1	Re

Centroceras internitens	0	0	0	0	0	0	0	0	1	1	0	0	Re
Ceramium brevizonatum var. *caraibicum*	0	0	0	1	0	0	0	1	0	1	1*	1	St
Ceramium cimbricum f. *flaccidum*	1	0	1	0	0	0	0	0	0	0	1*	1	St
Ceramium corniculatum	0	0	0	0	0	0	1	1	1*	0	0	1*	
Ceramium cruciatum	1	1	1	1*	1*	1	1	1*	1	0	1	1	St
Ceratodictyon intricatum	0	0	0	0	0	0	1	0	0	0	0	1	St
Ceratodictyon planicaule	0	1	1	0	0	0	0	0	0	0	1	0	St
Ceratodictyon scoparium	0	0	0	0	0	0	0	1*	0	0	0	0	St
Ceratodictyon variabile	1	1	1	0	0	1	0	0	0	1	0	1	St
Champia parvula	1	0	0	0	0	0	0	0	0	0	0	0	Re
Chondria baileyana	0	0	0	0	1	0	0	0	1	0	0	0	Ho
Chondria cnicophylla	0	0	0	0	0	0	0	1	0	0	0	0	Ho
Chondria dasiphylla	0	0	0	0	0	0	0	0	0	0	1	0	
Chondria floridana	1	0	1	1	1	1	1	0	0	0	0	0	
Chondria leptacremon	0	1	0	0	0	0	0	1	0	1	0	0	Ho
Digenea simplex	1	0	1	1	0	1	1	1	0	1	0	1	
Fosliella sp.	0	0	1	0	0	0	0	0	0	0	0	0	
Gayliella flaccida	0	0	1	0	0	0	0	0	1	0	0	1	St
Griffithsia schousboei	0	1	0	0	0	0	0	0	0	0	0	0	
Gritfitsia sp.	0	0	0	0	0	0	0	1	0	0	0	0	
Herposiphonia bipinnata	1	1	1	0	1	1	1	0	0	1	1	1	St
Herposiphonia cf. *parca*	0	0	0	1	0	0	1	0	0	0	1	1	St
Herposiphonia pecten-veneris	0	0	0	0	0	0	1	0	0	0	0	0	St
Herposiphonia secunda	1	1	1	0	1	1	1	1*	1	1	1	1	St
Herposiphonia tenella	0	0	0	0	1	0	1	1	1	0	0	0	St
Jania capillaceae	0	0	1	1	0	1	1	1	0	1	1	0	Ho, Re
Jania pumila	1	1	1	1	1	1	1	0	1	1*	1	1	Ho
Laurencia caraibica	0	0	1	0	0	0	0	0	0	0	0	0	
Lophosiphonia cristata	1	1	1	1	1	1	1	1	1	1	1	1	St
Lophosiphonia obscura	1	0	1	1	0	0	0	0	1	1	0	1	St
Neosiphonia gorgoniae	1	0	1*	1	1	0	0	1	1	1	0	1*	Re
Neosiphonia sertularioides	0	0	0	0	1	0	0	0	0	0	0	1	St
Neosiphonia sphaerocarpa	0	0	0	1	0	0	0	0	0	0	0	0	St
Neosiphonia subtilissima	0	0	1	0	0	0	0	0	0	0	0	0	St
Parviphycus setaceus	0	0	1	0	0	1	0	0	0	0	0	0	St, Re
Parviphycus trinitatensis	1	1	1	1	1	1	1	1*	1	1	1	1	St, Re

Polysiphonia binneyi	1*	0	0	0	0	0	0	1	0	1*	1	1	
Polysiphonia denudata	1	1	1	0	0	1	1	1	1	1	0	1	
Polysiphonia havanensis	0	1	1	1	0	1	1	1	1	1	1	1	
Polysiphonia howei	1	0	1	1	0	1	1	0	0	1	0	0	St
Polysiphonia opaca	1	0	1	0	0	0	0	0	0	0	0	0	
Polysiphonia pseudovillum	0	0	0	0	0	0	0	0	0	1	0	0	St
Polysiphonia scopulorum var. *villum*	1	1	1	1	0	1	1	1	1	1	1	1	St
Pterocladiella sanctarum	0	1	1	0	1	1	0	1	1	1	1	1	
Spyridia sp.	0	0	0	1	0	0	0	0	0	0	0	0	
Subtotal (51 taxa)	20	16	28	19	15	18	21	22	20	23	19	26	
**Chlorophyta**													
Bryobesia johannae	0	0	1	1	0	1	0	0	0	0	0	0	
Chaetomorpha sp.	0	0	0	0	0	0	0	0	0	1	0	0	
Chalmasia antillana	1	0	0	1	0	0	0	0	0	0	0	0	
Cladocephalus sp.	0	0	1	1	0	1	0	0	0	0	0	0	
Cladophora laetevirens	0	0	1	0	0	0	0	0	0	0	0	0	
Cladophora liniformis	1	0	1	0	0	0	1	0	0	1	0	0	
Cladophora vagabunda	0	1	0	0	0	0	0	0	0	0	0	0	St
Dictyosphaeria cavernosa	0	0	0	0	0	0	0	0	0	0	1	0	
Dictyosphaeria ocellata	1	0	0	0	1	0	0	0	0	0	0	0	
Enteromorpha sp.	0	0	1	0	0	0	0	0	0	0	0	0	
Halimeda goreaui	0	0	0	1	0	0	0	0	0	0	0	0	Fr
Parvocaulis polyphysoides	1	0	0	1	0	0	0	0	0	0	0	0	
Parvocaulis pusillus	1	0	0	0	0	0	0	0	0	0	0	0	
Penicillus capitatus	1	1	1	1	0	0	1	0	0	1	0	1	
Ulva flexuosa subsp. *paradoxa*	0	1	1	0	0	0	0	1	0	0	0	0	
Subtotal (15 taxa)	6	3	7	6	1	2	2	1	0	3	1	1	
**Phaeophyceae**													
Canistrocarpus cervicornis	0	0	0	0	0	1	0	0	0	0	0	0	Re
Cladosiphon occidentalis	0	0	0	0	0	0	0	1	0	0	0	0	
Dictyerpa stage of *Padina*	1	1	1	1	1	1	1	1	1	1	1	1	
Dictyota caribaea	0	0	0	0	1	0	0	0	0	0	0	1	Re
Dictyota friabilis	0	0	1	0	0	1	0	0	0	0	0	0	
Dictyota sp.	0	0	1	1	0	1	0	0	0	0	0	0	Fr
Ectocarpus sp.	0	0	0	0	0	0	0	0	0	0	0	1	

Feldmannia mitchelliae	0	0	1	0	0	0	1	0	1	0	0	0	
Hincksia onslowensis	1	0	1	0	0	0	0	0	0	0	0	0	
Lobophora variegata	1	0	1	0	0	0	0	0	0	0	0	0	Re
Padina sp.	0	0	1*	1	0	0	0	0	0	0	0	0	
Sphacelaria novae-hollandiae	0	0	0	0	0	1	0	0	0	0	0	0	St
Sphacelaria tribuloides	1*	0	0	0	0	0	0	0	0	1	0	0	St
Sphacelaria sp.	1	1	1*	1	1	1	0	1	1	1	0	1	
Subtotal (14 taxa)	5	2	8	4	3	6	2	3	3	3	1	4	
TOTAL (86 taxa)	35	24	48	33	24	32	28	29	26	33	24	34	

Supplementary Table S5 | Mean values (± standard deviation) of the morphological characters of *Parviphycus trinitatensis* and *Polysiphonia scopulorum* var. *villum* growing in surrounding live and dead corals covered by TA from *Orbicella faveolata* under the experimental conditions in six experimental treatments (T) and on two sampling dates. Raw data. See Fig. 1 and text for details

Morphological characters	***Parviphycus trinitatensis***		***Polysiphonia scopulorum* var. *villum***	
	**May**	**August**	**May**	**August**
**Distance between erect axes (µm)**				
T1	964.0 (± 478.8)	755.6 (± 355.1)	481.7 (± 180.9)	439.0 (± 85.1)
T2	716.7 (± 395.6)	849.4 (± 361.7)	518.1 (± 167.4)	455.0 (± 144.4)
T3	794.5 (± 420.5)	883.2 (± 521.6)	598.0 (± 165.3)	367.3 (± 182.6)
T4	740.9 (± 435.0)	630.4 (± 251.0)	588.5 (± 200.4)	519.7 (± 206.2)
T5	601.1 (± 260.8)	826.6 (± 327.0)	705.5 (± 186.9)	616.7 (± 182.6)
T6	575.8 (± 363.0)	790.5 (± 345.4)	716.4 (± 228.5)	569.9 (± 232.9)
**Height of erect axis (µm)**				
T1	4120 (± 1800)	3200 (± 1800)	2600 (± 1900)	2200 (± 1500)
T2	3500 (± 2500)	3100 (± 2300)	2200 (± 1500)	2700 (± 2600)
T3	3600 (± 2400)	3700 (± 1900)	2500 (± 1700)	2500 (± 1100)
T4	3400 (± 2100)	4000 (± 2300)	2300 (± 1900)	2600 (± 1500)
T5	4200 (± 2700)	3300 (± 2000)	3200 (± 2400)	3100 (± 2000)
T6	4200 (± 2400)	3500 (± 2500)	2000 (± 1400)	2300 (± 1600)
**Diameter of prostrate axis (µm)**				
T1	182.0 (± 120.0)	130.0 (± 41.0)	90.5 (± 20.8)	70.1 (± 30.2)
T2	132.0 (± 41.0)	110.0 (± 40.4)	81.0 (± 20.2)	70.3 (± 20.0)
T3	136.0 (± 21.0)	130.0 (± 41.0)	80.3 (± 20.6)	70.4 (± 30.6)
T4	122.0 (± 30.5)	110.0 (± 40.3)	181.5 (± 20.5)	80.5 (± 30.2)
T5	150.0 (± 20.5)	180.0 (± 111.0)	90.2 (± 10.4)	110.2 (± 40.5)
T6	132.0 (± 40.7)	110.0 (± 40.2)	90.6 (± 20.1)	80.15 (± 30.0)
**Distance between rhizoids (µm)**				
T1	1092.6 (± 1049.8)	1762.8 (± 931.8)	599.9 (± 418.3)	334.0 (± 119.0)
T2	2055.1 (± 1424.5)	1825.1 (± 1000.7)	338.7 (± 186.4)	366.9 (± 157.3)
T3	1671.6 (± 834.3)	1661.6 (± 1055.0)	547.5 (± 544.7)	400.8 (± 200.7)
T4	1689.4 (± 981.6)	1719.5 (± 1842.8)	280.5 (± 107.0)	512.8 (± 400.7)

T5	2408.4 (± 1165.9)	1799.5 (± 1135.3)	456.1 (± 464.1)	338.0 (± 178.4)
T6	797.1 (± 286.1)	1613.9 (± 826.2)	545.1 (± 548.5)	553.3 (± 585.2)
**Formation of new ramets (%)**				
T1	35.0 (± 28.5)	5.5 (± 11.8)	26.1 (± 14.0)	1.8 (± 5.5)
T2	16.3 (± 21.1)	2.9 (± 7.6)	19.1 (±16.8)	7.1 (± 18.9)
T3	40.7 (± 23.8)	25.8 (± 21.1)	40.7 (± 21.5)	0.0 (± 0.0)
T4	15.0 (± 13.2)	10.7 (± 15.3)	0.0 (± 0.0)	13.9 (± 17.8)
T5	53.3 (± 17.6)	13.3 (± 21.7)	25.0 (± 25.0)	0.0 (± 0.0)
T6	5.0 (± 11.1)	12.5 (± 19.4)	0.0 (± 0.0)	0.0 (± 0.0)

Supplementary Table S6 | Results of two-way ANOVA of the effects on the morphological characters of *Parviphycus trinitatensis* and *Polysiphonia scopulorum* var. *villum* growing in surrounding live and dead corals covered by TA from *Orbicella faveolata* under the experimental conditions (factors: treatment (T1-T6) and date (May and August)). ns = not significant

**Morphological characters**	**df**	**MS**	**F**	**p**	**Conclusion**
**Distance between erect axes**					
Parviphycus trinitatensis					
Treatment	5	84.30	2.02	0.076	ns
Date	1	111.20	2.66	0.104	ns
Treatment x Date	5	105.80	2.53	0.029	significant
Polysiphonia scopulorum var. *villum*					
Treatment	5	138.50	9.52	0.000	T5 ~ T6 > T1, T2, T3, T4
Date	1	360.20	24.74	0.000	May > August
Treatment x Date	5	29.70	2.04	0.070	ns
**Height of erect axis**					
Parviphycus trinitatensis					
Treatment	5	0.32	0.98	0.430	ns
Date	1	1.32	4.02	0.040	May > August
Treatment x Date	5	0.56	1.73	0.120	ns
Polysiphonia scopulorum var. *villum*					
Treatment	5	0.28	1.12	0.350	ns
Date	1	0.27	1.07	0.300	ns
Treatment x Date	5	0.17	0.68	0.640	ns
**Diameter of prostrate axis**					
Parviphycus trinitatensis					
Treatment	5	0.01	4.46	0.000	T1 ~ T5 > T2, T3, T4, T6
Date	1	0.00	0.00	0.973	ns
Treatment x Date	5	0.00	2.32	0.043	significant
Polysiphonia scopulorum var. *villum*					
Treatment	5	0.00	3.48	0.004	T1, T6 ~ T5 > T2, T3, T4
Date	1	0.01	49.85	0.000	May > August
Treatment x Date	5	0.00	1.67	0.141	ns

**Distance between rhizoids**					
Parviphycus trinitatensis					
Treatment	5	490.80	3.49	0.000	T6 < T1, T2, T3, T4, T5
Date	1	1.10	0.01	0.005	May < August
Treatment x Date	5	317.40	2.26	0.930	ns
Polysiphonia scopulorum var. *villum*					
Treatment	5	81.72	1980.66	0.143	ns
Date	1	8.19	1.66	0.683	ns
Treatment x Date	5	115.35	0.17	0.041	significant
**Formation of new ramets**					
Parviphycus trinitatensis					
Treatment	5	1213.00	3.70	0.006	T1, T5 ~ T3 > T2, T4, T6
Date	1	3628.60	10.90	0.001	May > August
Treatment x Date	5	724.30	2.20	0.068	ns
Polysiphonia scopulorum var. *villum*					
Treatment	5	490.00	2.70	0.027	T1, T2, T4, T5 ~ T3 > T6
Date	1	3243.70	18.20	0.000	May > August
Treatment x Date	5	948.70	5.30	0.000	significant
